# Men’s willingness to pay for prostate cancer screening: a systematic review

**DOI:** 10.1186/s13643-020-01522-3

**Published:** 2020-12-09

**Authors:** Hiro Farabi, Aziz Rezapour, Najmeh Moradi, Seyed Mohammad Kazem Aghamir, Jalil Koohpayehzadeh

**Affiliations:** 1 Department of Health Economics, School of Health Management and Information Sciences, Iran University of Medical Sciences, Tehran, Iran; 2 Health Management and Economics Research Center, School of Health Management and Information Sciences, Iran University of Medical Sciences, Tehran, Iran; 3 Health Management and Economics Research Center, Iran University of Medical Sciences, Tehran, Iran, Iran University of Medical Sciences, Tehran, Iran; 4 Urology Research Center, Tehran University of Medical Sciences, Tehran, Iran; 5 Preventive Medicine and Public Health Research Center, Psychosocial Health Research Institute. Community and Family Medicine Departmentm School of Medicine, Iran University of Medical Sciences, Tehran, Iran

**Keywords:** Willingness to pay, Contingent valuation method, Prostate cancer screening, Early detection, Systematic review

## Abstract

**Background:**

This study aimed to review studies on willingness to pay (WTP) for prostate cancer screening.

**Methods:**

This systematic-review was conducted based on the Preferred Reporting Items for Systematic Reviews guidelines. By searching six-health-database, WTP studies on prostate cancer screening using contingent valuation method published in English until March 2020 were included and those with unavailable full-text and inadequate quality-assessment scores were excluded. Smith checklist was used for the quality assessment. Extracted WTPs were converted to US dollar in 2018 using exchange rate parity and net present value formula to make comparison. Factors’ effect was assessed by vote counting.

**Results:**

Six final studies published after 2006 reported above 70% Smith checklist items needed to be considered in contingent valuation study reports. Seven factors have positive effects on WTP. The reported WTP value varied from 11$ to 588$ in Japan and Germany, respectively.

**Conclusion:**

WTP for prostate cancer screening was positive among all studied men. The results of factors’ effect assessment showed that better understanding prostate cancer risks or screening tests and factors such as age, income, family history of cancer, hospitalization history, and educational level have positive effects. Moreover, prostate-specific antigen history, health insurance, employment, and subject’s health assessment received less attention.

The results’ generalization to all countries is not applicable because there are no studies for low- and middle-income countries.

**Systematic review registration:**

PROSPERO 2020 CRD42020172789

**Supplementary Information:**

The online version contains supplementary material available at 10.1186/s13643-020-01522-3.

## Background

Prostate cancer is one of the most common cancers, which is also known as the second most dangerous cancer among men [1]. It is estimated that one out of six men at the age of 65 years old and above will develop prostate cancer during his lifetime [2].

Prostate cancer imposes a significant economic burden on the health system. The annual cost per every prostate cancer including the cost of hospitalization and treatment is reported to be between 106.7 and 179 million euros in European countries, 9.862 billion dollars in the USA, 103.1 million Canadian dollars, and 101.1 million Australian dollar between 1993 and 1994 [3]. In most cancers, the cost of cancer treatment gradually increases since 12 months prior to death, which then increases sharply in the last 2 months before death [4]. Prostate cancer is no exception and its treatment costs increase along with the disease’s progress. In the USA in 2006, the mean cost of diagnosing and treating prostate cancer at the early stages of this disease was calculated as 10,612 dollars, which reached 33,691 dollars in the last year of patients’ life [3].

Despite the rising rates of prevalence and mortality in some developing countries, the existing evidence suggests that mortality rate has declined in some high-income countries. Accordingly, this reduction is attributed to screening, the use of other early diagnosis methods, and improved treatment [5]. Notably, early detection of cancer is considered as an important factor in controlling the disease, reducing the cost of treatment, and increasing the rate of survival; therefore, it is necessary to highlight the significance of health education in developing countries where people have inadequate knowledge on the screening methods [6]. Early successful detection of prostate cancer also helps in the elimination or reduction of treatment costs and money saving via treating fewer men with advanced stages of prostate cancer and metastasis [7].

The effectiveness of prostate cancer screening has been investigated in different studies [8, 9]. A free of charge screening for prostate cancer in Tyrol region of Austria resulted the decreased rate of mortality up to 40–50% [10]. Moreover, a recent study that investigated the screening test for prostate cancer in Europe, suggested that mortality rate has relatively decreased by early detection, despite the high rate of the prevalence of this cancer [11].

The health care financial resources are scarce and decision on allocating treatment and prevention is an excessively complex and challenging process [12]. In this regard, determination of the costs and benefits of screening can help in prioritization and optimal allocation. The level of participation in screening programs to benefit estimation is also crucial. If people are not willing to participate in screening programs, it would not be possible to screen them. Also, on the other hand, people’s high willingness to participate can lead to additional contribution in financing part of the screening costs in the form of out of pocket payments, which consequently reduces the costs of implementing screening programs for health policymakers. Through early detection, screening prevents a reduction in the quality of life and reduces the risk of death, hence it is theoretically expected that people would welcome screening. However, prediction is not enough to make a policy, and quantitative evidence is needed. So, a monetary measure is needed to assess people’s willingness to participate in screening. As a result, using the willingness to pay (WTP), as a monetary estimation, individual appraisal of available programs and goods can be measured and a quantitative estimation of their WTP for screening can be provided [13]. Therefore, WTP can be regarded as mental value of goods for customers, because they want to pay such money for the good. Moreover, this helps health system policymakers in achieving an approximation for consumers’ willingness to participate in financing preventive care services through WTP estimation.

Given the role of the stated preference studies to provide more accurate and policy relevant results, this study aimed to investigate studies conducted on examining men’s WTP for prostate cancer screening. Accordingly, the factors affecting the WTP were also reviewed as much as possible.

## Methods

### Data

#### Search for studies

This systematic review was conducted in terms of the Preferred Reporting Items for Systematic Reviews (PRISMA) guidelines. Various health databases including Pubmed, Embase, Scopus, Global Health, Google Scholar, and Web of Science were systematically searched. Also, government reports, discussion papers, gray literature or references of the extracted articles, and WHO database were searched to prevent loses of important information or further related studies. The keywords were selected using the US National Library of Medicine’s Medical Subject Headings (Mesh), which were as follows: (“Prostate-Specific Antigen” AND “Mass Screening” AND “Willingness to pay” AND “Patient Acceptance of Health Care”).

Search strategies are shown in Additional file 1.

### Inclusion and exclusion criteria

#### Inclusion criteria

This systematic review included those contingent valuation studies that investigated men’s WTP for hypothetical or specific prostate cancer screening test using contingent valuation method (CVM) and were published until March 2020. The language of the studies was restricted to English.

#### Exclusion criteria

Studies using other techniques to estimate WTP, whose full text was not available, and studies that did not obtain enough score in quality assessment were excluded.

### Quality assessment

The quality of the final studies was assessed using Smith checklist [14]. This checklist proposes a guideline in health subjects to illustrate some important points concerning the conduct of contingent valuation (CV) studies. In addition, it includes 34 questions in four categories as follows: CV development and context, CV scenario description, CV reporting and results, CV validity, and reliability. In order to score the study’s quality assessment, completely reported questions would be signed with “YES” and those that were not covered in the reviewed articles would be signed with “NO.” The “Yes” and “No” scores are considered as “1” and “0,” respectively.

### Data analysis

After searching for resources, Endnote V.9 software was used to organize the included studies. At first, the studies whose title and abstract did not meet the inclusion criteria were removed. Thereafter, two reviewers (HF, NM) read the full text of the remaining studies and extracted the key specifications designed. The collected data were entered into independent sheets and their differences, and any disagreement between the researchers were investigated by Cohen’s kappa and then resolved by a third researcher (AR). The accepted standards to interpret kappa coefficient (KC) have been proposed as follows: poor for KC ≤ 0, slight for KC = .01–.20, fair for KC = .21–.40, moderate for KC = .41–.60, substantial for KC = .61–.80 = substantial, and almost perfect for KC = .81–1 [15]

In order to assess the risk of bias, any disagreement between researchers was discussed by team members.

Adjusting the values obtained from health economic study is necessary in different study for making them comparable [16]. In the present study, in order to compare the results, WTP index obtained in different studies was converted to US dollar value in 2018 using exchange rate parity and net present value formula. In cases where the year was not reported, the year of publication of the study was used as the basis for currency conversion [13].

In order to decide on performing meta-analysis, heterogeneity among studies was also assessed.

Heterogeneity statistic interpreting is as follows: 25% for low heterogeneity, 50% for medium heterogeneity, and 75% and above for high heterogeneity [17].

Moreover, vote counting was used to identify the factors that more often affected prostate cancer screening WTP as follows:

Different factors were identified and their effect on WTP was also assessed. The factors were categorized by their signs and significance including significant positive, significant negative, or non-significant positive and non-significant negative effects on WTP in included study and then gave a vote to each factor. Finally, the votes were counted and each factor that obtained at least three votes or factors have the same sign and significance results in all the reviewed articles was considered as an affecting factor on prostate cancer screening WTP.

## Results

### Search result

**Fig. 1:**
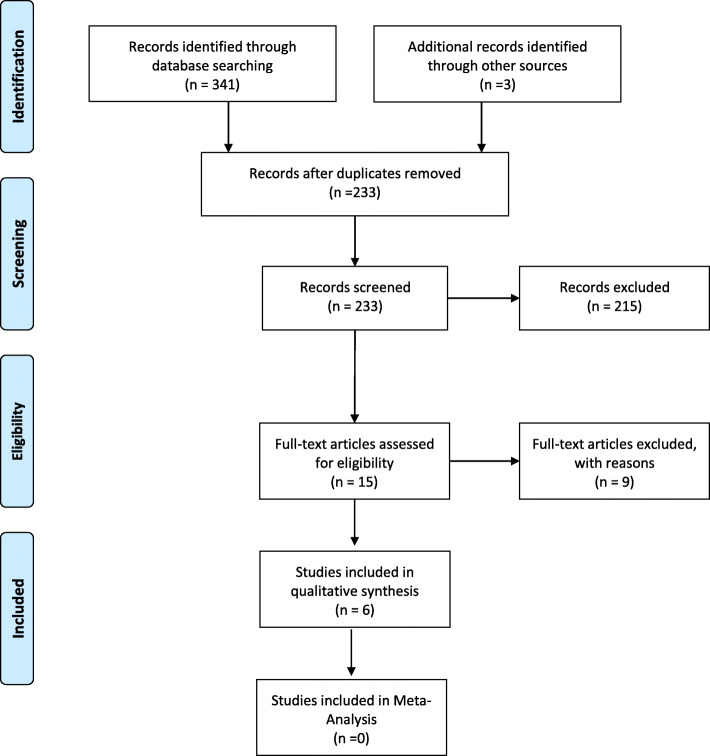
PRISMA flow diagram for study selection

A total of 344 related studies were identified in the initial search (Fig. 1). There was no disagreement in search process. 327 duplicate or irrelevant articles were removed, and 18 related articles were then entered into the next stage for performing more evaluation. Also, 12 studies were excluded due to following results: their full text was not available (3), they did not meet the inclusion criteria (7), and they reported inadequate or inappropriate information (2). Finally, six articles were included in this study and then reviewed.

Agreement between interviewers (HF, NM) was perfect. The Cohen’s kappa coefficient in selection studies was 0.85 (Additional file 3). Also, according to accepted standards to interpret kappa, agreement was perfect. Totally, there were two disagreements between the two reviewers, which were resolved by the third reviewer (AZ).

### Quality assessment result

The final studies were scored based on the Smith checklist items, and the percentage of scores obtained was calculated. Results are summarized in Table 1, showing that the scores of four studies [19–22] were above 75%. The other articles’ scores were between 65% and 74%.

**Table 1:** The quality of methodology of studies

Checklist of what should be reported in published CV studies	Neumann et al. 2010 [18]	Yasunaga 2008 [19]	Yasunaga et al. 2006 [20]	Yasunaga et al. 2011 [21]	Pedersen et al. 2011 [22]	Mayer et al. 2018 [23]
**CV development and context**						
Country where the CV survey has been conducted and health care financing details	Yes	Yes	Yes	Yes	Yes	Yes
Focus—methodological or policy	Yes	Yes	Yes	Yes	Yes	Yes
Specificity of questionnaire (part of wider survey)	Yes	Yes	Yes	Yes	Yes	Yes
Details of other measures of QoL incorporated	No	Yes	Yes	Yes	Yes	No
Scenario development	Yes	Yes	Yes	Yes	Yes	Yes
Welfare measure (WTP or WTA)	Yes	Yes	Yes	Yes	Yes	Yes
**CV scenario description**						
Intervention(s)	Yes	Yes	Yes	Yes	Yes	Yes
Partiality (single good or close substitutes)	Yes	Yes	Yes	Yes	Yes	Yes
Outcomes (health status, probability, and time)	Yes	Yes	Yes	Yes	Yes	Yes
Non-outcomes (information, care, other)	Yes	Yes	Yes	Yes	Yes	Yes
Payment vehicle	Yes	No	No	No	Yes	No
Presentation of uncertainty/risk	Yes	Yes	Yes	Yes	Yes	No
Survey period	No	Yes	Yes	Yes	Yes	Yes
Time period for WTP	No	No	No	No	No	No
Question/elicitation format	Yes	Yes	Yes	Yes	Yes	Yes
**CV reporting and results**						
Method of data collection	Yes	Yes	Yes	Yes	Yes	Yes
Type of respondent	Yes	Yes	Yes	Yes	Yes	Yes
Sample size	Yes	Yes	Yes	Yes	Yes	Yes
Response rate	Yes	Yes	Yes	Yes	Yes	Yes
Type of outcomes incorporated (use, option, or externality value)	Yes	Yes	Yes	Yes	No	Yes
Duration of interview/length of questionnaire	No	Yes	Yes	Yes	Yes	Yes
WTP values (results of the studies)	Yes	Yes	Yes	Yes	Yes	Yes
Transformation of values from one context/time to another	No	No	No	No	No	No
Price year	No	No	No	No	No	No
Currency	Yes	Yes	Yes	Yes	Yes	Yes
Cost of intervention	No	No	No	No	No	No
Cost–benefit ratio	No	No	No	No	No	No
Time period used in analysis	No	No	No	No	No	No
**CV validity and reliability**						
Tests for bias—order effect, starting point, range, interviewer, strategic	Yes	Yes	Yes	Yes	Yes	No
Statistical analysis performed	Yes	Yes	Yes	Yes	Yes	Yes
Assessment of zero/high bids	Yes	No	Yes	Yes	Yes	Yes
Distributional issues consider	Yes	Yes	Yes	Yes	No	Yes
Validity tests	Yes	Yes	Yes	Yes	Yes	No
Reliability tests	Yes	Yes	Yes	Yes	Yes	No
Rate of responses	74%	76%	79%	79%	76%	65%

It is noticeable that although the scores were acceptable in quality evaluation, the items of “Payment vehicle” and “Time period for” received less attention in final studies. The items of “Transformation of value options from one context/time to another,” “Price year,” “Time period used in analysis,” and “Cost of intervention” were not provided in any of the included studies. While to have the best estimation of WTP, this item needs to be provided.

### Characteristics of the selected included studies

The assessment shown in Table 2 indicated that all the finally selected studies were published in the last 14 years (2006 and beyond), which were conducted in developed countries [18–23]. Summary of the questionnaires or scenarios were reported in all of the studies.^1^ Accordingly, some studies [18, 19, 21–23] used double-bounded dichotomous-choice approach and one of them [20] used payment card technique for the estimation of WTP. Five studies used prostate-specific antigen test in their scenarios, except one study that used hypothetical testing [23]. Populations in all of studies were men without symptoms and signs of cancer except one study [18] that was performed on prostate cancer patients. Participants’ response rate was reported in all the studies, which was ranged from 33% [19] to 96% [23]. Respondents replied through online and computer-based questionnaires in all the studies. Different factors affecting on WTP have been examined in the studies including demographic factors, level of income, level of education, a history of testing, a family history of prostate cancer, history of any disease, and some other factors. Some studies [19, 21, 22] have also examined the effect of additional information such as information on screening test, its side effects, and prostate cancer risks, as the main subject, on WTP results. The results are summarized in Table 3.

**Table 2:** Description of WTP study characteristics

Author/year	Country	Study aim	Respondents (***N*** = sample size)	Response rate	Examination test	CVM techniques	Regression model	Significant factors	Number of scenarios	Statistical measure
**Neumannet al. 2010** [18]	USA	Assesses how much people would pay for a laboratory test that predicted their future disease status.	688 men without symptoms—age not presented	0.96%	Predictive test	Double-bounded, dichotomous-choice approach	Logistic regression and maximum likelihood regression	Age (negative) Household Income (positive) Risk score (positive) Education (negative) Gender (positive)	2	Median $263 for perfect and mean $622 for the perfect prostate cancer test in risk disease 25%
**Yasunaga 2008** [19]	Japan	Estimating the willingness to pay (WTP) for prostate cancer screening with prostate-specific antigen (PSA).	400 men without symptoms aged 50–59	0.33%	PSA	A double bound dichotomous choice approach	Weibull regression analysis	Age (positive) Annual household income (positive) Family history of cancer (positive)	1	The mean WTP was ¥1670 ($15.2)
**Yasunaga et al. 2006** [20]	Japan	Verifying this hypothesis that having sufficient information will reduce men’s desire for screening.	137 men without symptoms aged 40–59	0.36%	PSA	Payment Card	Categorical regression analysis	Age (positive) Household income (positive) Hospitalization History (positive)	1	The mean WTP for prostate-specific antigen screening was $18.90
**Yasunaga et al. 2011** [21]	Japan	Comparing the WTP between well-informed and ill-informed men to pay for PSA screening.	1800 men without symptoms aged 50–69 years	0.50%	PSA	Double-bound dichotomous choice method.	Weibull regression analysis	Household income (positive) history of receiving PSA screening (positive)		The average WTP was significantly greater in group 1 than in group 2 ($31.1 vs. $25.1,)
**Pedersen et al. 2011** [22]	Denmark	Assessing the impact of public and private health care services, and the extent to which negative information on the PSA-test influences the perceptions of the screening programmed.	1535 men without symptoms aged 50–70 years	0.40%	PSA	Double bounded dichotomous choice	multiple regression	Household income (positive) Employment (negative) Prior PSA-test (negative) User fees (positive)	3	Full sample—excluding protesters (DDK) Public provision and low information = 85.3
**Mayer et al. 2018** [23]	German	Achieving insight into men’s attitudes in genetic testing for PCa.	4699 prostate cancer patients	0.70%	PSA	Double bounded dichotomous choice	logistic regression	Self-reported economic situation (positive) Family history (positive) Education (positive)	3	Up to 500 Euro

**Table 3:** Summery description of WTP study characteristics

Study characteristics	References
**Published year** • 2006–2010 • 2011–2015 • 2016–2020	[19, 20] [18, 21, 22] [23]
**Country** • Japan • Germany • USA • Denmark	[19–21] [23] [18] [22]
**Population** • Prostate cancer patient • Men without symptoms	[23] [18–22]
**Data collection** • Web and Internet-based questionnaire	[18–23]
**Respondence rate** • 30–50% • 51–70% • 71–90% • 91–100%	[19–22] [23] [18]
**Examination test** • Prostate-specific antigen • Detective test	[19–23] [18]
**WTP technical** • Double-bound dichotomous choice method • Payment card	[18, 19, 21–23] [20]
**WTP value (mean, median)** • 11$ (1670 ¥) • 13$ (18.90$) • 25$ (31.1$) • 69$ (85.3DDK)^1^ • 491$ (622$) • 588$ (500€)	[19] [20] [21] [22] [18] [23]

^1^Given the different WTPs was reported in this study, we used the highest reported WTP in our analysis

### WTP for prostate screening test

**Fig. 2:**
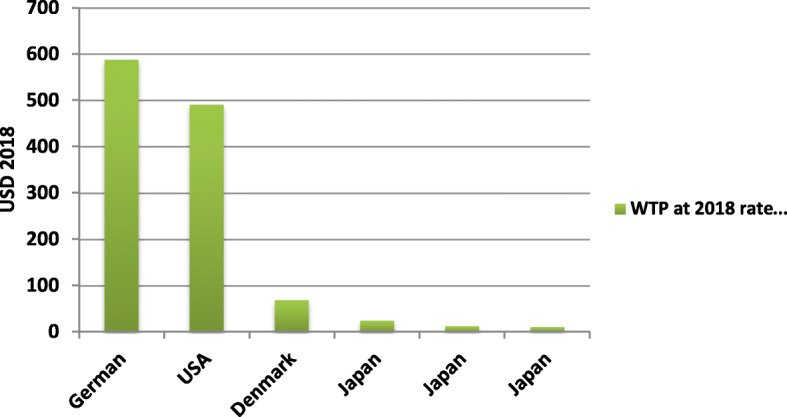
The WTP for prostate cancer screening in USD

In the assessment of the extracted WTP statics, studies have shown a different range of mean value of WTP that they were not comparable because of differences of currencies among different studied countries. Comparing them was possible after converting in specific discount. Because the last study was for 2018, all studied WTP were converted to US dollar value in 2018 and discounted at discount rate 3% by net present value formula. Figure 2 presents WTP for prostate cancer screening. As shown, the highest value, i.e., $ 588, was reported in Germany and the lowest value, i.e., $ 11.3, was reported in Japan [19].

### Studies’ heterogeneity

Heterogeneity is an important challenge in WTP literature [24]. This review had some criteria that made a high heterogeneity including type of information in scenarios, number of scenarios, using model for estimation, type of screening test, type and number of factors investigated in scenarios, type of study population and sample size, and methods for estimating CVM that are summarized in Table 2.

Furthermore, heterogeneity in effect size was assessed by use of statistic heterogeneity (*I*^2^). The *I*^2^ statistic describes the percentage of variations across studies that were due to heterogeneity, rather than chance [25].

Calculations showed that in df = 5, *Q* = 548.994, (*P* < 0.00001), *I*^2^ static equals to 99% (Additional file 3). These results show the presence of a high heterogeneity, and in this situation, conclusions of a meta-analysis are compromised; therefore, meta-analysis was not performed.

### Factors affecting WTP for prostate cancer screening

#### Characteristics

Understanding the affecting factors on WTP for prostate cancer screening is important in financing mass screening policies and for future research design.

Different factors affecting on WTP and their significance were examined in all the final studies. Age and income were the most frequently investigated factors reported among the studies [18–23]. Different models were used in the final studies to estimate WTP and assessing factor affecting on them, including logistic regression [18, 23], Weibull regression analysis [19, 21], categorical regression analysis [20], multiple regression [22], and maximum likelihood regression [23]. There was no limitation in the selection of the used model.

Some studies [18, 22] examined a wider range of factors affecting WTP, and the other studies examined a limited number of factors affecting WTP. It seems that there is not a unique producer about the included factors affecting on WTP.

#### Vote counting

The sign of affecting factors was not the same in all the studies. Due to the incompatibility of the coefficients in different studies, vote counting was used to conclude about the factors affecting prostate cancer screening WTP (Table 4). The factors affecting on WTP were specified and the results show that age (5 to 0)^2^, income (6 to 0), and type of information (3 to 0) were the most influencing factors on WTP, so that their effects were proven, respectively. In addition, some factors including educational level, family history of cancer, hospitalization history, and cancer risk have significant effects (2 to 0), because they have the same sign and significance results in all the reviewed articles. The significant effect was not proven for the other factors.

**Table 4:** Factors affecting on WTP

Author	Neumannet al. 2010 [18]	Yasunaga 2008 [19]	Yasunaga et al. 2006 [20]	Yasunaga et al. 2011 [21]	Pedersen, et al. 2011 [22]	Mayer et al. 2018 [23]	Vote
Age	**↑↑**	**↓**	**↑↑**	**↑↑**	**↑↑**	**↑↑**	(5 to 0)
History of PSA screening	–	**↓**	–	**↑↑**	**↓↓**	–	[1 to 1]
Family history of cancer	–	**↑↑**	–	**↑**	**↑↑**	–	(2 to 0)
Income	**↑↑**	**↑↑**	**↑↑**	**↑↑**	**↑↑**	**↑↑**	(6 to 0)
Type of information sheet	–	**↓**	**↑**	**↑↑**	**↑↑**	**↑↑**	(3 to 0)
Hospitalization history	–	–	**↑↑**	**↑↑**	–	–	(2 to 0)
Subject assessment of health	–	**↓**	**↑**	–	–	–	0
Education	**↑↑**	–	–	–	–	**↑↑**	(2 to 0)
Lethal prostate cancer	–	–	–	–	**↓↓**	**↑↑**	[1 to 1]
Employment	–	–	–	–	**↑↑**	–	(1 to 0)
Cancer risk	**↑↑**	–	–	–	**↑↑**	–	(2 to 0)
Health insurance	–	–	–	–	**↓↓**	–	(0 to 1)

↑↑Positive and significant effect. ↓↓ Negative and significant effect**.** ↑ **Positive and non-significant effect.** ↓ Negative and non-significant effect

## Discussion

From an ethical point of view, there is no barrier to involve patients in important decisions on providing care services for them; rather, it is a way to improve the quality of services and to “prevent unnecessary and costly medical interventions” [26]. This research has systematically reviewed all studies that investigated WTP for prostate cancer screening. The results of six selected studies showed that WTP for prostate cancer screening among men was always positive and its value was higher in some countries such as Germany and the USA [18, 23], while it was lower in Japan [19].

Furthermore, the reviews of these studies that have examined the effect of information on WTP showed that information have a positive and significant effect [18, 21, 22]. So, obtaining more information on screening test such as its side effects and prostate cancer risk did not almost reduce people’s WTP, even in cases with a negative impact [19, 21] on the WTP; it was non-significant and WTP’s value did not reach zero. Generally, men’s preference was screening and paying for it.

Age is the main affecting factor with the proven positive effect. As shown that prostate cancer is an aging cancer, men’s willingness is to pay attention to rise with aging.

Also, income and having a good financial status were identified as positive factors affecting WTP for prostate cancer screening in all the included studies. Having a high income make a high WTP, hence in designing screening programs, policymakers could consider this issue and provide different screening packages to different income deciles.

Experiencing any disease or being at the risk of prostate cancer, as well as a family history of prostate cancer [18, 19, 22] and history of hospitalization [20, 21] have positive and significant effects on WTP and motivates patients’ relatives and family members to undergo prostate cancer tests. In other word, experiencing a disease encourages men’s doing something that keeps them away from risk. It shows that people are in search of peace of mind and they are willing to pay for it when they are under stress condition due to family members’ involvement in prostate cancer.

Educational level was also identified as a positive factor on WTP [18, 23]. The results show that men’s with a higher educational level have higher willingness to pay for prostate cancer screening. Higher level of education may make better understanding of health care importance on the quality of life or may be men have more complete information on the devastating effects of the disease because of the higher level of study.

The effect of history of PSA test in two studies [21, 22] was not consistent. Generalization of the results and conclusion about it were not possible because the assessed studies were not sufficient. So, the inconsistency between the results needs to be further investigated in future studies before generalizing the results.

There was not enough evidence on the other factors including health insurance, employment, and subject assessment of health to conclude, and extra research is needed to decide about their effects on WTP.

Furthermore, it became obvious that the effect of differences between patient and healthy men on WTP have not been analyzed, so far. It can be considered as an important reason of different WTP values between these studies.

Generalizing the result to all countries is not applicable, because all studies were performed in developed countries and no studies were found for low- and middle-income countries. Therefore, it is necessary for developing countries to design new studies with their special features.

All the reviewed studies used the Internet and web-based methods to send and complete their questionnaires. It seems that using face-to-face methods to collect data increases the reliability of study results.

An important advantage of this study was the quality assessment of the final studies. Although the final studies had acceptable scores in qualitative evaluation, this assessment showed that in designing scenarios of WTP, the factors related to “payment vehicle” and “time period” in smith checklist have been less considered. While the type of payment or financing for prostate cancer screening and periods of repeating the tests can have effects on WTP, which are necessary to be considered in the future studies’ scenario designing.

Although the number of studies entered into final analysis was small, but it is possible to make valid conclusions on some of affecting factors and to set a reasonable interval for WTP. However, this review had heterogeneity in some factors including type and numbers of scenarios, the used model of estimation, information varieties in scenarios, sample size, and type of population (healthy subjects and prostate cancer patients). Assessing effect size heterogeneity showed a high heterogeneity between the included studies. Because in a high heterogeneity, the conclusions of a meta-analysis are compromised, and meta-analysis was not performed in this review. So, vote counting was used to assess the affecting factors.

## Conclusion

Given the growing trend of cancer patients and the related treatment costs, the health systems must seriously consider the utilization of early detection and screening methods, to identify people with cancer in a timely manner for the purpose of reducing the related costs. The results of this study indicated the positive WTP for prostate cancer screening among all the studied men. In addition, the assessment of factors affecting the WTP showed that obtaining more information on screening test and prostate cancer risk make a higher WTP. The effect of age on WTP is positive and men would have a higher WTP in older age. Furthermore, education, history of cancer, and history of hospitalization have positive and significant effects on WTP.

Also, the positive and significant effect of income was proven in all studies. So, policymaker can use it as a main option to provide different screening package for different income deciles in designing screening program. The impacts of a health insurance, employment, subject assessment of health, and history of PSA screening have received less attention, and there were no fully known effects for them.

All the reviewed studies used the Internet and web-based methods to send and complete their questionnaires. It seems that using face-to-face methods to collect data increases the reliability of study results.

Generalizing the result to all countries is not applicable, because there are no studies for low- and middle-income countries. Therefore, to determine the value of prostate cancer screening, besides the utilization of the results of the reviewed studies, it is necessary to conduct further research in low- and middle-income countries.

## Supplementary Information

**Additional file 1**

**Additional file 2**

**Additional file 3**

## Abbreviations

WTP: Willingness to pay
CVM: Contingent valuation method
CV: Contingent valuation
PSA: Prostate-specific antigen
KC: Kappa coefficient

## Footnotes

1 Considering the length of scenarios; they are given in Additional file 2 instead of placing them in the data extraction table.

2 5 is the number of studied positive and significant effects and 0 the number of negative and significant effect.
