# Supplementary material for: Men’s willingness to pay for prostate cancer screening: a systematic review
Source: Syst Rev. 2020 Dec 9;9:290. doi: 10.1186/s13643-020-01522-3 (PMC7727201; doi:10.1186/s13643-020-01522-3)
Supplement: Supplementary file 1 — Additional file 1 [file 13643_2020_1522_MOESM1_ESM.docx]

| **Pubmed** | Search: **("Prostate-Specific Antigen"[MH]OR "gamma Seminoprotein" [Title/Abstract] OR "Prostate Specific Antigen" [Title/Abstract] OR "PSA"[ Title/Abstract]) AND("Prostatic Neoplasms"[MH] OR "Prostate Neoplasms"[ Title/Abstract] OR "Prostate Neoplasm"[ Title/Abstract] OR "Prostatic Neoplasm"[ Title/Abstract] OR "Prostate Cancer"[ Title/Abstract] OR "Prostate Cancers"[Title/Abstract] OR "Prostatic Cancer"[ Title/Abstract] OR "Prostatic Cancers"[Title/Abstract])AND("Mass Screening" [MH] OR "Screening" [Title/Abstract] OR "Screenings" [Title/Abstract]) AND ("Patient Acceptance of Health Care" [MH]OR "willingness To pay"[Title/Abstract]) AND ("1996/01/01"[Date - Publication] : "2020/03/30"[Date - Publication])**  **OR**  Search: **("Prostate-Specific Antigen"[MH]OR "gamma Seminoprotein" [Title/Abstract] OR "Prostate Specific Antigen" [Title/Abstract] OR "PSA"[ Title/Abstract]) AND("Prostatic Neoplasms"[MH] OR "Prostate Neoplasms"[ Title/Abstract] OR "Prostate Neoplasm"[ Title/Abstract] OR "Prostatic Neoplasm"[ Title/Abstract] OR "Prostate Cancer"[ Title/Abstract] OR "Prostate Cancers"[Title/Abstract] OR "Prostatic Cancer"[ Title/Abstract] OR "Prostatic Cancers"[Title/Abstract])AND("Mass Screening" [MH] OR "Screening" [Title/Abstract] OR "Screenings"[Title/Abstract]) AND ("Patient Acceptance of Health Care" [MH]OR "willingness To pay"[Title/Abstract]) AND ("1990/01/01"[Date - Publication] : "2020/03/30"[Date - Publication])** |
| --- | --- |
| **Embase** | (**'prostate specific antigen'**:ti,ab OR **'gamma seminoprotein'**:ti,ab OR **'psa gene'**:ti,ab) AND (**'prostate tumor'**:ti,ab OR **'prostate neoplasms'**:ti,ab OR **'prostate cancer'**:ti,ab) AND (**'mass screening'**:ti,ab OR **'screening'**:ti,ab) AND (**'patient attitude'**:ti,ab OR **'willingness to pay'**:ti,ab) AND [1996-2020]/py |
| **WOS** | TS=  (“Prostate-Specific Antigen”)  AND  TS=  (“Prostatic Neoplasms” OR “Prostate Neoplasms” OR “Prostate Neoplasm” OR “Prostatic Neoplasm” OR “Prostate Cancer” OR “Prostate Cancers” OR “Prostatic Cancer” OR “Prostatic Cancers”)  AND  TS=  (“Mass Screening” OR “Screening” OR “Screenings”)  AND  TS=  (“Patient Acceptance of Health Care” OR “willingness To pay” )  *Indexes=SCI-EXPANDED, SSCI, A&HCI, CPCI-S, CPCI-SSH, BKCI-S, BKCI-SSH, ESCI, CCR-EXPANDED, IC Timespan=1996-2020* |
| **Scopus** | **TITLE-ABS-KEY** (**"**Prostate-Specific Antigen**"** OR **"**gamma Seminoprotein**"** OR **"**Prostate Specific Antigen**"** OR **"**hK3 Kallikrein**"** OR **"**Semenogelase**"** OR **"**Kallikrein hK3**"** OR **"**Seminin**"** OR **"**PSA**"**) AND **TITLE-ABS-KEY** (**"Prostatic Neoplasms" OR "Prostate Neoplasms" OR "Prostate Neoplasm" OR "Prostatic Neoplasm" OR "Prostate Cancer" OR "Prostate Cancers" OR "Prostatic Cancer" OR "Prostatic Cancers")** AND **TITLE-ABS-KEY ("Mass Screening" OR "**Screening**"** OR **"**Screenings**"**) AND **TITLE-ABS-KEY** (**"**Patient Acceptance of Health Care**"** OR **"**willingness To pay**"** ) |
| **Proquest** | [("Prostate-Specific Antigen") AND ab("Prostatic Neoplasms" OR "Prostate Neoplasms" OR "Prostate Neoplasm" OR "Prostatic Neoplasm" OR "Prostate Cancer" OR "Prostate Cancers" OR "Prostatic Cancer" OR "Prostatic Cancers") AND ab("Mass Screening" OR "Screening" OR "Screenings") AND ab("Patient Acceptance of Health Care" OR "willingness To pay")](https://search.proquest.com/recentsearches.recentsearchtabview.recentsearchesgridview.scrolledrecentsearchlist.checkdbssearchlink:rerunsearch/434C1DDDE9CF4FE2PQ/None?t:ac=RecentSearches)Limits applied |
| **Cochrane** | Prostate-Specific Antigen AND (“Prostate Cancer” OR “Prostatic Cancer”) AND (“Mass-Screening” OR “Screening” OR "screenings") AND (“Patient Acceptance of Health Care” OR “willingness to pay”)  with Cochrane Library publication date from Jan 1996 to Apr 2020 |
