# Supplementary material for: Men’s willingness to pay for prostate cancer screening: a systematic review
Source: Syst Rev. 2020 Dec 9;9:290. doi: 10.1186/s13643-020-01522-3 (PMC7727201; doi:10.1186/s13643-020-01522-3)
Supplement: Supplementary file 2 — Additional file 2 [file 13643_2020_1522_MOESM2_ESM.docx]

**Appenndix (2):**

**Yasunaga H, (2011)**

**APPENDIX 1: PSA SCREENING INFORMATION SHEETS (ORIGINALLY IN JAPANESE)**

**SHEET A**

The prostate gland is a male organ about the size of a chestnut at the base of the bladder. Approximately 10 000 men die of prostate cancer every year in Japan. Although men in their 40s rarely contract prostate cancer, the incidence rate increases after 50 years of age and increases

further thereafter.

Prostate cancer screening measures prostate-specific antigen (PSA) levels in the blood. The percentage of positive results in PSA screening is about 8%. If PSA levels exceed the standard value, further examination will be recommended. This examination will consist, initially, of transrectal ultrasonography to visualize the prostate gland, with a device inserted through the anus. The examination could also include a biopsy, which involves removing several samples of cells by inserting a needle into the prostate. PSA screening is expected to reduce the risk of dying of prostate cancer by approximately 20%.

**SHEET B**

(The following information was added to the information contained in sheet A.)

The PSA test has several limitations. The test is subject to false-negative results, in which the measured PSA levels are lower than the standard value, despite the presence of cancer. False-negative results occur at a rate of 20%-30%. Thus, the presence of cancer cannot be ruled out by a single PSA test in 20%-30% patients with prostate cancer. Men with negative results still need to be continuously followed up.

About 40% of men who undergo biopsy for high PSA levels will be diagnosed with prostate cancer. In contrast, 60% of men who undergo biopsy will have done so on the basis of a falsepositive result (ie, they will be diagnosed as cancer free). Biopsies can be complicated by bleeding (ie, hematuria, rectal bleeding, or hematospermia) or infection in about 15% of cases. Most symptoms are mild and heal spontaneously; only 0.7% of patients require hospitalization to treat biopsy complications.

Death from biopsy is very rare, at 1/200 000. Some cases of prostate cancer progress so slowly that the cancer might not end up being the cause of death, even if left untreated. Such cancer is called clinically insignificant cancer and accounts for approximately 10% of screening-detected cancers.

**APPENDIX 2: WTP QUESTION (ORIGINALLY IN JAPANESE)**

This survey aims to study the economic viability of cancer screening. All the questions are hypothetical, and there are no correct or incorrect answers to the questions. Please answer the question with due consideration that paying for the screening will result in a reduction in the amount of money available for other goods or services.

Q1. Assume that you can undergo PSA screening. Would you be willing to pay $X for the test?

Yes or no.

Q2. Then, would you be willing to pay $Y? Yes or no. given the controversy surrounding the use of mammography in the United States, and its similarities as a screening test to PSA,1-3 a U.S. population would be willing to pay the same or even more, given the same evidence. Evidence-based medicine does not seem to influence an autonomous patient’s desire to know his prostate cancer status, regardless of the clinical value of such information. What we as clinicians do with that information when counseling a patient is of far greater importance.

**Yasunaga H, (2006)**

**Information Sheet A**

The prostate gland is a male organ about the size of a chestnut at the base of the bladder. While males in their forties rarely contract prostate cancer, morbidity increases after they reach 50 years of age and increases further thereafter. Prostate cancer has no obvious subjective symptoms in the initial stages. Prostate cancer screening measures prostate specific antigen (PSA) level in blood. If PSA level exceeds the standard value, a closer examination will be recommended. This examination will consist, initially, of transrectal ultrasonography to visualize the prostate gland, with a device inserted through the anus. The examination may also include a biopsy, which involves removing six samples of cells by inserting a needle into the prostate. The examination may be complicated by bleeding or infection in a small number of cases. The cancer detection rate for the screening amounts to 0.93%.

**Information Sheet B**

(The following information was added to the information contained in Information Sheet A)

The accuracy of the PSA test is not perfect. The test is subject to false negative results, where PSA levels are measured to be lower than the standard value in spite of the presence of cancer. False negative results occur at a rate of 10-20%. Accordingly, the presence of cancer cannot be ruled out 100%, even if the PSA value is lower than the standard value, and individuals need to be continuously followed up. Thirty to forty percent of cases that undergo biopsy for high PSA levels will be diagnosed with cancer. On the contrary, 60–70% of cases that undergo biopsy will be diagnosed as free from cancer or to have a false positive result Some cases of prostate cancer progress so slowly that it may not cause death, even if left untreated. Such cancer is called latent cancer. However, latent cancer is rarely detected by PSA screening. It is apparent that the detection rate of prostate cancer will increase as the number of subjects undergoing PSA screening increases. However, it has not been clearly demonstrated to date whether this will lead to lowering of the mortality rate from prostate cancer.

**WTP question**

Assuming that you can get a PSA screening for prostate cancer once a year on a chargeable basis, what is the maximum amount you would be willing to pay for the screening? This survey aims to study academically the economic viability of cancer screening. There are no correct or wrong answers to the survey.

Please answer the question with due consideration to the fact that paying for the screening will result in a reduction in the amount of money which can be used for other goods or services.

Options: $0, $5, $10, $20, $30, $50, $70, $100

**Pedersen LB & et,al, (2011)**


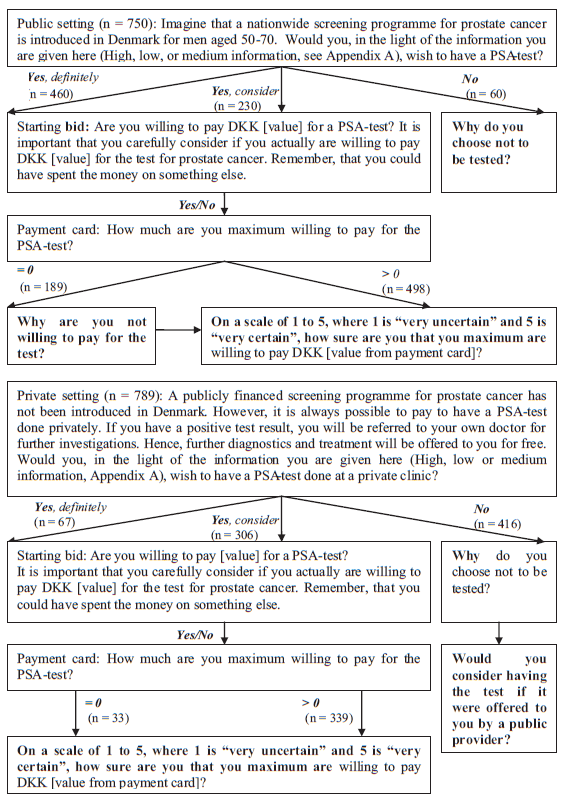


**Yasunaga H, (2008)**

**Table 1**

**Information sheets and WTP questionnaire (Originally in Japanese)**

**Information Sheet A**

The prostate gland is a male organ about the size of a chestnut at the base of the bladder.

While males in their forties rarely contract prostate cancer, morbidity increases after they reach 50 years of age and increases further thereafter.

Prostate cancer has no obvious subjective symptoms in the initial stages.

Prostate cancer screening measures prostate specific antigen (PSA) levels in the blood.

If PSA levels exceed the standard value, further examination will be recommended. This examination will consist, initially, of transrectal ultrasonography to visualize the prostate gland ,with a device inserted through the anus. The examination may also include a biopsy, which involves removing several samples of cells by inserting a needle into the prostate. The examination may be complicated by bleeding or infection in a small number of cases.

The cancer detection rate for the screening is 0.93%.

**Information Sheet B**

(***The following information was added to the information contained in Information Sheet A)***

The accuracy of the PSA test is not perfect. The test is subject to false negative results, where PSA levels are measured to be lower than the standard value in spite of the presence of cancer. False negative results occur at a rate of 10–20%. Accordingly, the presence of cancer cannot be ruled out 100% even if the PSA value is lower than the standard value, and individuals need to be continuously followed up.

Thirty to 40 percent of cases that undergo biopsy for high PSA levels will be diagnosed with cancer. On the contrary, 60–70% of cases that undergo biopsy will be diagnosed as being free from cancer or having a false positive result.

Some cases of prostate cancer progress so slowly that the cancer may not end up being the cause of death, even if left untreated. Such cancer is called latent cancer. It is apparent that the detection rate of prostate cancer will increase as the number of subjects undergoing PSA screening increases.

However, it has not been clearly demonstrated to date whether this will lead to lowering of the mortality rate from prostate cancer.

**WTP Question**

This survey aims to study academically the economic viability of cancer screening. There are no correct or wrong answers to the survey. Please answer the question with due consideration to the fact that paying for the screening will result in a reduction in the amount of money which can be used for other goods or services.

**Q1.** Assume that you can get a PSA screening for prostate cancer once a year on a chargeable basis. Would you be willing to pay ¥X for the screening? Select one of the following five choices:

(1) Yes, definitely; (2) Yes, probably; (3) I am not sure; (4) No, probably

not; (5) No, definitely not.

**Q2.** Then, would you be willing to pay ¥Y? Select one of the following five choices:

1. Yes, definitely; (2) Yes, probably; (3) I am not sure; (4) No, probably not; (5) No, definitely not

**Mayer M & et, al (2019)**

The main outcome variables were patients’ *interest in genetic testing, willingness-to-pay for a genetic test* and *willingness-to-recommend a genetic test* to male relatives, assessed by three questions in the annual follow-up questionnaire.

For the two questions “If there were a genetic test for prostate cancer, would you take such a test?” and “Would you recommend a genetic test to your relatives, if it helped to assess their risk for developing prostate cancer?”

three response categories (“definitely”, “rather yes” and “rather no”) were provided. For the third question “If you had to pay for the genetic test yourself, how much would you be willing to pay?” five response categories were provided (“< 200€”, “200–500€”, “500–1000€”, “1000–2000€” and “> 2000€”). For the analysis, responses were dichotomized into the categories yes (definitely yes and rather yes) versus no and < 500€ versus ≥ 500€.

**Neumann PJ & et, al, (2012)**

**Sample Scenario**

Imagine that you go to the doctor for your regular check up. Your doctor tells you that you are healthy but that someone your age has a 25% chance of some day getting………………. The people in red represent the number out of 100 that might get …………... As you may know, is a disease of ………….. Most people who get ………..disease is over age …..

The doctor tells you that a new blood test is available that will tell you know whether or not you will one day develop ……... The test is 100% accurate. You will have to pay for the test yourself because insurance does not pay for it. Assume for this scenario that you cannot prevent the disease from occurring. For people who get the disease, there are treatments available that may help.

Would you want to take the test if it cost you $50?

🚹🚹🚹🚹🚹🚹🚹🚹🚹🚹

🚹🚹🚹🚹🚹🚹🚹🚹🚹🚹

🚹🚹🚹🚹🚹🚹🚹🚹🚹🚹

🚹🚹🚹🚹🚹🚹🚹🚹🚹🚹

🚹🚹🚹🚹🚹🚹🚹🚹🚹🚹

🚹🚹🚹🚹🚹🚹🚹🚹🚹🚹

🚹🚹🚹🚹🚹🚹🚹🚹🚹🚹

🚹🚹🚹🚹🚹🚹🚹🚹🚹🚹

🚹🚹🚹🚹🚹🚹🚹🚹🚹🚹

🚹🚹🚹🚹🚹🚹🚹🚹🚹🚹
