# Supplementary material for: Men’s willingness to pay for prostate cancer screening: a systematic review
Source: Syst Rev. 2020 Dec 9;9:290. doi: 10.1186/s13643-020-01522-3 (PMC7727201; doi:10.1186/s13643-020-01522-3)
Supplement: Supplementary file 3 — Additional file 3 [file 13643_2020_1522_MOESM3_ESM.docx]

1. **Kappa statistics**


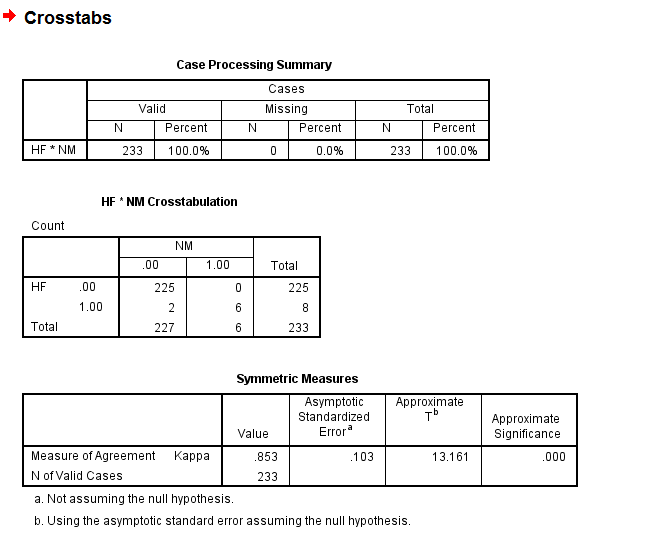


1. **Heterogeneity I^2^ Statistic**

| Author | year | WTP | event | sample size | outcome | se | var | w* | w*es | w*(es^2) | w^2 |
| --- | --- | --- | --- | --- | --- | --- | --- | --- | --- | --- | --- |
| Yasunaga H | 2008 | 11 | 400 | 1200 | 0.333333333 | 0.016667 | 0.000278 | 3600 | 1200 | 400 | 12960000 |
| Yasunaga H | 2006 | 13 | 137 | 380 | 0.360526316 | 0.030802 | 0.000949 | 1054.015 | 380 | 137 | 1110946.774 |
| Yasunaga H | 2011 | 25 | 1800 | 3596 | 0.500556174 | 0.011798 | 0.000139 | 7184.009 | 3596 | 1800 | 51609983.72 |
| Pedersen | 2011 | 69 | 1535 | 3901 | 0.393488849 | 0.010043 | 0.000101 | 9913.877 | 3901 | 1535 | 98284954.65 |
| Neumann PJ | 2012 | 491 | 688 | 1906 | 0.360965373 | 0.013762 | 0.000189 | 5280.285 | 1906 | 688 | 27881408.45 |
| Mayer M | 2019 | 588 | 4699 | 7296 | 0.644051535 | 0.009395 | 8.83E-05 | 11328.29 | 7296 | 4699 | 128330064.1 |
|  |  |  |  |  |  |  |  | 38360.47 | 18279 | 9259 | 320177357.7 |
| k=6 | 6 |  |  |  |  |  |  |  |  |  |  |
| df=5 | 5 |  |  |  |  |  |  |  |  |  |  |
|  |  |  |  |  |  |  |  |  |  |  |  |
| Q | 548.9443 |  |  |  |  |  |  |  |  |  |  |
| I^2 | 99.08916 |  |  |  |  |  |  |  |  |  |  |
|  |  |  |  |  |  |  |  |  |  |  |  |
|  |  |  | es | 0.476506 |  |  |  |  |  |  |  |
|  |  |  | SEes | 0.005106 |  |  |  |  |  |  |  |
|  |  |  | CI | 0.466499 | 0.486513373 |  |  |  |  |  |  |
